# Turning the tide on turnover: The impact of empowering leadership on the work-family spillover of managers

**DOI:** 10.1371/journal.pone.0287674

**Published:** 2023-08-10

**Authors:** Naseer Abbas Khan, Waseem Bahadur, Robin Maialeh, Natayla Pravdina, Maria Akhtar

**Affiliations:** 1 Department of Industrial Economics and Project Management, South Ural State University, Chelyabinsk, Russia; 2 College of International Cooperation and College of Business, Xi’an International University, Xi’an, Shanxi, China; 3 Research Institute for Labour and Social Affairs, Prague, Czech Republic; 4 School of Business, UCT Prague, Prague, Czech Republic; 5 Malik Firoz Khan Noon Business School, University of Sargodha, Punjab, Pakistan; KFUPM: King Fahd University of Petroleum & Minerals, SAUDI ARABIA

## Abstract

The aim of this study is to examine the direct and indirect relationships between empowering leadership (EL), work-family spillover and manager turnover intentions, and to explore the moderating impact of perceived organizational support (POS) on these relationships. The study collected data from 220 participants—middle-level managers and their immediate subordinates working in hotels and tourism-related enterprises in central China. The results highlighted a significant relationship between EL and work-family positive spillover (WFPS) and manager turnover intentions, whereas the mediating effect of work-family negative spillover (WFNS) was found to be insignificant. The results further indicated that POS exerted a significant moderating impact on the association between EL and manager turnover intentions, and a significant mediating impact on WFPS. The study also determined that neither the mediating impact of WFNS nor the relationship between EL and WFNS was affected by POS. The study provides a unique perspective on empowering leadership based on the Conservation of Resources theory, and contributes to the understanding of its effects on manager turnover intentions.

## 1. Introduction

Interest in the challenges associated with the work-family balance has recently experienced a significant increase in terms of research attention [1]. The study focuses on the tourism and hospitality sectors due to their particularly high employee turnover rates [2]. Further, the hospitality and tourism industries present particular difficulties for managers and employees with respect to maintaining the balance between their work and family responsibilities due to the demanding working hours and challenging targets that are characteristic of these sectors [3, 4]. While attention has been devoted to work-family spillover as a phenomenon in its entirety, limited research has been conducted on the distinction between work-family positive spillover (WFPS) and work-family negative spillover (WFNS) in the context of leadership and its impact on followers. Many of the arguments presented in the literature fail to clearly differentiate between the effects of work and family. For example, it is common for individuals to transfer their emotions, behavior and attitudes from one domain to the other [5].

In contrast, a number of management researchers have argued that individuals who attempt to balance multiple tasks may become stressed and overwhelmed if they have insufficient time or energy to fulfill all their responsibilities [6]. Researchers are, therefore, faced with the challenge of examining the various forms of spillover that occur between work and family, taking into account factors such as stress and anxiety [7]. Leadership research has played a critical role in our understanding of how different leadership styles are able to impact the work-family conflict [8]. Despite the research already conducted on the impact of leadership styles, e.g. servant leadership and ethical leadership, on work-family outcomes [9], further research is required in order to identify the underlying mechanisms that connect empowering leadership (EL) and the intentions of managers. EL is a leadership style that involves delegating authority and providing employees with the autonomy to make decisions and to take action [10]; it is based on the belief that individuals who are given the power to make decisions and act upon them are more motivated and committed to achieving their goals, which leads to higher levels of job satisfaction and organizational success [11].

Sharma and Kirkman [12] determined that managers in the hospitality and tourism industries empower their subordinates since they are unable to either be completely in control or make all the decisions required. The literature on EL focuses mainly on the work-family spillover of employees [13], whereas it devotes insufficient attention to the effect of EL on the work-family spillover and intentions of managers (e.g. turnover intentions). Work-family spillover refers to the transfer of experiences, attitudes and behavior from the work domain to the family domain or vice versa [14]. This may be either positive or negative, depending on the nature of the spillover. Previous research has concentrated primarily on employee spillover in work-family studies without the thorough consideration of positive and negative spillover. In addition, there is a lack of literature on leadership studies devoted to the examination of how the two types of work-family spillover (positive and negative) impact the turnover intentions of managers.

The overall aim of this study is to investigate how EL affects the turnover intentions of managers in the Chinese tourism sector, aimed at which the study has two specific objectives. The first objective is to examine the impact of work-family spillover (both positive and negative) as mediators. The second objective is to analyze the role of perceived organizational support (POS) as a moderator in the direct and indirect relationship between EL and the turnover intentions of managers. POS refers to an employee’s perception of the extent to which their organization values their contributions and cares about their well-being [15]. A previous study determined that POS reduced employee turnover intentions, work-family conflicts and job burnout [16]. In addition, it attenuated the link between emotional intelligence and conflicts between work and the family.

In this overall context, the study intends to contribute to the literature on leadership in the tourism sector by providing both theoretical and practical insights. Using the COR theory as its foundation, the study presents a moderated mediation model and tests the integration of EL with WFNS and WFPS aimed at enhancing the understanding of the relationship between EL and the turnover intentions of managers. This is followed by an investigation of the underlying mechanisms, e.g. WFNS and WFPS, that influence the relationship between EL and the turnover intentions of managers. In addition, the study intends to deepen our understanding of the impact of EL on managers and their work-family balance, particularly in the context of the tourism sector, and to examine the mediating influence of work-family spillover and the moderating role of POS in the relationship between EL and turnover intentions. The study collected data applying the time-lag approach over two time waves and includes the performance of multi-level analysis aimed at highlighting the importance of empowering executives in terms of expressing their intentions at work, and draws attention to the role of POS as a moderator in the relationship between EL and the turnover intentions of managers. Finally, the study emphasizes the significance of examining the underlying mechanisms of work-family spillover in the context of EL and turnover intentions.

## 2. Theory and hypothesis

### 2.1 COR theory

The concept of leadership empowerment has already been widely discussed in the literature as a positive form of leadership behavior, particularly in terms of relieving employee stress. However, it can also be argued that the benefits of empowering leadership so extend to the managers themselves. In COR theory, Hobfoll provides a framework for understanding the ways in which employees respond to leadership and how they cope with stress. Resources can be classified in terms of three categories: material resources (e.g. money and housing), social resources (e.g. family and organizational support) and psychological resources (e.g. intentions and behavior). WFNS can be considered to be a stressor that acts to deplete these resources and render individuals more susceptible to future stressors. WFNS refers to the transfer of negative experiences, attitudes, and behavior from the work domain to the family domain and vice versa. WFNS can be particularly harmful to employees and their families in terms of increased levels of stress, burnout and conflict. Conversely, WFPS refers to the transfer of positive experiences, attitudes and behavior from the work domain to the family domain and vice versa [17]. The scarcity of resources often leads to more aggressive responses than in cases where there is a surplus of resources. However, certain resources are able to assist in the recovery of lost resources. This highlights the importance of considering the impact of leadership empowerment not only on employees but also on managers themselves, as well as the potential role of different types of resources in terms of mitigating the effects of stress and negative spillover.

In the COR theory, Hobfoll [18] postulates that stress can lead to the depletion of the psychological resources of individuals, which potentially results in negative outcomes. This theory is in alignment with the model presented in this study, which integrates the concept of EL with manager intentions via the work and family domains, thereby replenishing psychological resources. Adhering to the tenets of the COR theory, we consider EL to be a catalyst that serves to enhance psychological resources and, in turn, to improve employee behavioral outcomes, including positive impacts on work and the family [19]. In contrast, according to the COR theory, when individuals experience a loss of resources, they attempt to replenish these resources or adopt measures aimed at preventing their further depletion [18].

**Fig 1:**
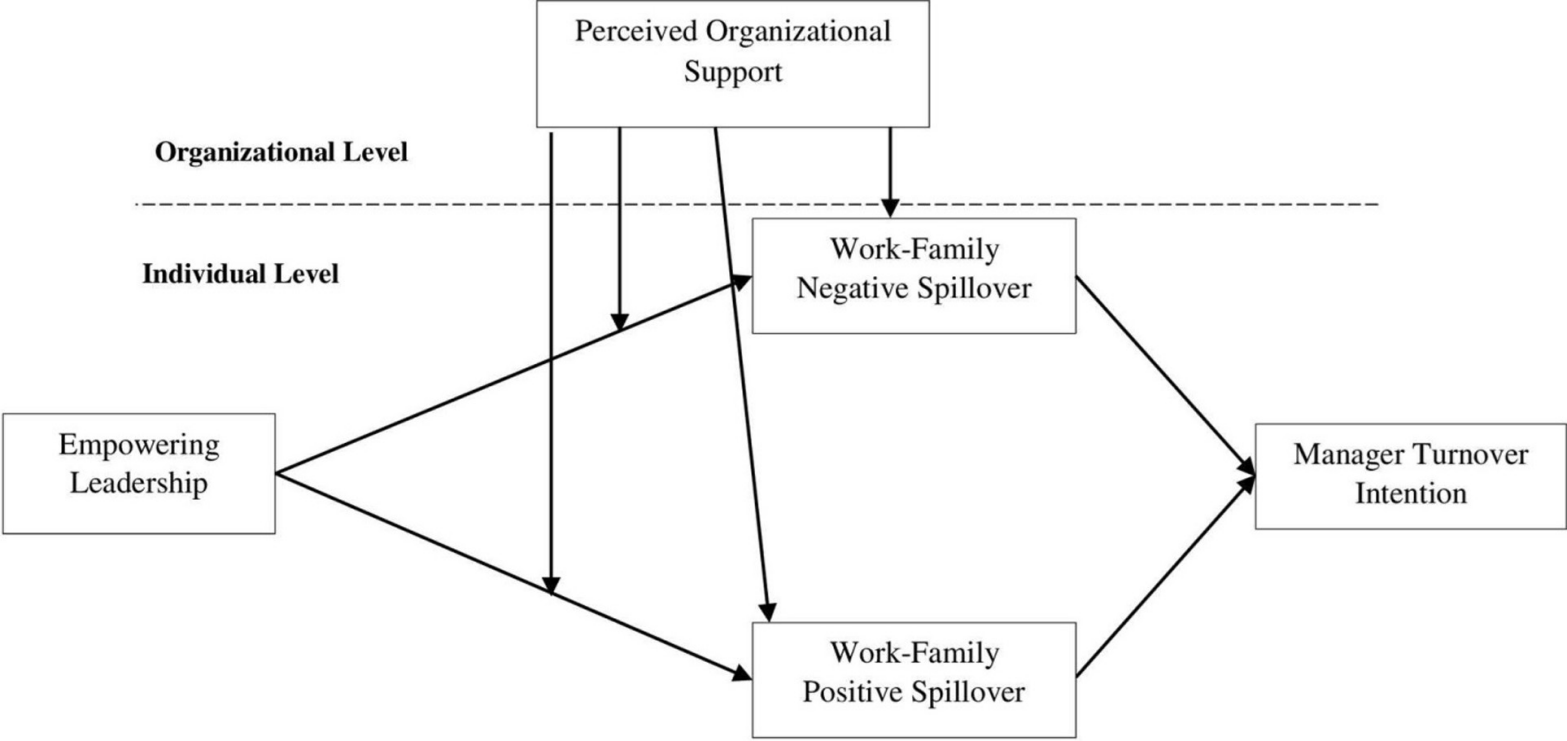
Research model.

Our hypothesis proposes that EL reduces intention-to-turnover stress, resulting in a harmonious work-family spillover and a reduction in the extent of negative spillover. Historically, the relationship between leadership and employee performance has been linked with work-family conflict, emotional exhaustion, anxiety and low levels of self-control [20, 21]. Our model, based on the COR theory, is consistent with these arguments (as shown in Fig 1), and also expands the application of the theory in several ways. Firstly, empowering behavior fosters motivation and confidence, which help to conserve the individual’s resources. Secondly, the impact of work-family spillover on the individual’s happiness and stress levels has the potential to exert an impact on their internal resources.

### 2.2 EL, work-family spillover and turnover intentions

The concept of EL has been examined extensively and frequently introduced in the organizational setting [22] aimed at addressing the rapidly changing environments of organizations [23]. Previous research has established that EL offers substantial benefits for both organizations and employees [10] using a multilevel modeling paradigm [24]; it leads to elevated levels of creativity, job performance, job satisfaction [25, 26] and job crafting [27]. This article applies the work-home resource frame work so as to enhance the effect of EL on WFNS.

Individuals who possess certain resources are better equipped to manage work-related stress, and those who acquire personal resources from work-family resources are less likely to leave their jobs [28]. Family resources may serve as a mediator in the relationship between work resources and work-family spillover. The researchers found that reinforcing leadership through employee engagement leads to increased job satisfaction. Autonomy, autonomy-encouraging conditions, mutual decision-making, leadership by example and equal opportunities can be fostered via the strengthening of leadership [29]. Under such EL conditions, employees attain their goals through enhanced creativity at both the individual and team levels [30], as well as via improved job performance [31]. This leads to the following hypotheses.

**H1:** EL is negatively associated with the WFNS of managers.

**H2:** EL is positively associated with the WFPS of managers.

Organizations have substantial potential to assist their employees in terms of mitigating conflict and enhancing enrichment, thus resulting in enhanced levels of job satisfaction and performance [19]. It is possible to gain a deeper understanding of the underlying mechanisms related to the home-work interface via the merging of the spillover theory with role theories [13]. This study aims to formulate hypotheses concerning the impact of leadership empowerment on the work-family spillover effects (both positive and negative) of managers. Moreover, the study also attempts to examine the mediating role of work-family spillover in the relationship between EL and the turnover intentions of managers. In accordance with these objectives, the following hypotheses are proposed:

**H3:** Manager WFNS is a significant mediator in the relationship between EL and the turnover intentions of managers.

**H4:** Manager WFPS is a significant mediator in the relationship between EL and the turnover intentions of managers.

### 2.3 Moderating role of POS

The concept of perceived organizational support encompasses two factors: instrumental support and emotional support [32]. Previous research has indicated a positive relationship between perceived organizational support and job satisfaction, suggesting that perceived organizational support contributes to managerial engagement [19]. Conflicts between work and family are moderated by both professional engagement and perceived organizational support, which are closely inter-related [19]. It has been shown that perceptions of organizational support are able to foster positive attitudes and behavior towards the organization as a whole [32]. Numerous studies conducted with respect to Western populations, Brazilian populations and professionals from various industries have demonstrated the buffering effect of perceived organizational support in terms of mitigating work-family conflict [33]. Moreover, perceived organizational support fosters a sense of accountability and engagement with concern to the success of the organization [34], promotes a positive work environment and performance approach [32], and exerts a positive impact on mental health. The effective management of work-family conflict is a critical skill that managers should strive to develop both for their own benefit and that of their subordinates. In terms of support in this respect, organizations might provide project managers and other professionals with work-life balance training.

The concept of training initiatives aimed at empowering the workforce has been widely explored in previous studies. A study by Zhang and Zhou [35] highlights the importance of including both managers and non-management employees in such initiatives. In addition, the buffering hypothesis, as discussed [36], promotes the need for further research on the impact of various types of support (e.g. career and social support) in terms of reducing work-family conflict. This study aims to explore the moderating impact of perceived organizational support on the relationship between work-family stress and supportive factors. The following hypotheses are proposed aimed at demonstrating this effect.

**H5:** POS moderates the relationship between EL and WFNS in such a way that this relationship weakens as the level of POS increases and vice versa.

**H6:** POS moderates the relationship between EL and WFPS in such a way that this relationship is strengthened as the level of POS increases and vice versa.

POS plays a crucial role as a moderator in terms of shaping the indirect relationship between EL and the turnover intentions of managers via the work-family spill over mechanism. This study proposes the following hypotheses aimed at investigating the moderating effect of POS on the indirect relationship between EL and turnover via WFNS and WFPS.

**H7:** POS moderates the indirect relationship between EL and turnover intentions via the WFNS of managers in such a way that this indirect relationship weakens as the POS level increases and vice versa.

**H8:** POS moderates the indirect relationship between EL and turnover intentions via the WFPS of managers in such a way that this indirect relationship is strengthened as the POS level increases and vice versa.

## 3. Method

### 3.1 Data collection procedure

The study included the collection of data from hotels and tourism enterprises in Central China and involved approaching 360 potential respondents with the help of their respective human resources managers. The survey was administered through emails and WhatsApp numbers, and the final sample size consisted of 220 respondents. In order to gather the relevant data, a questionnaire was compiled using the seven-point Likert scale in English. Subsequently, the English questionnaire was translated into Chinese in order to facilitate the participation of middle management and their immediate subordinates, with the exception of the construct of POS. We obtained the relevant ethical approval from our institution’s ethics department, which granted us formal permission to collect survey responses from the participants. We provided participants with information on their consent and ensured that the objectives of the study were made clear to all the participants.

The first section of the questionnaire was aimed at gathering demographic information from the respondents, e.g. age, gender and marital status. Prior to the commencement of the survey, the respondents were informed that their information would be kept confidential and that their identities would be kept anonymous. The results of the survey indicated that 63% of the respondents were men, and the majority of participants (49%) were between the ages of 21 and 30, while 41% were between the ages of 31 and 40. On average, the respondents had tenure of 3.36 years in their current organization.

### 3.2 Measurement instrument

All the items included in each of the constructs in the study were rated on a seven-point Likert scale, which ranged from 1 (strongly disagree) to 7 (strongly agree). A 12-item scale developed by Ahearne and colleagues [27] was used to measure the EL construct. In addition, the study applied a 4-item scale developed by Grzywacz and Marks [17] to measure WFNS and an 11-item scale developed by Hanson and colleagues [37] to measure WFPS. The turnover intention was measured using a 4-item scale developed by Alsh and colleagues [38], which was originally designed to measure the turnover intentions of employees, with minor modifications to the item statements due to the study’s consideration of managers rather than employees. The study employed a 9-item scale to measure POS, as adopted from Robert Eisenberger an colleagues [39]. The effects of age, gender, education and tenure with the organizations were controlled in the study. The suitability of the individual scores to the level of the organization was also examined, with the results showing an average rwg (j) of 0.7, ICC (1) values of 0.40 and ICC (2) values of 0.92. The group effects were found to be statistically consistent with POS, and the ANOVA results revealed a significant organizational effect (p< 0.001).

## 4. Results

### 4.1 Correlation matrix

The results presented in Table 1 reveal that none of the correlations exceeded the corresponding square root of the Average Variance Extracted (AVE) in the diagonal cells of Table 1. These findings suggest that the discriminant validity values are satisfactory.

**Table 1:** Correlations matrix.

Variable	Mean	Std. Deviation	1	2	3	4	5	6	7	8	9
Gender	0.63	0.48	-								
Age	2.61	0.67	0.08	-							
Education	2.81	0.67	-0.01	0.27**	-						
Organizational tenure	3.36	1.56	0.00	-0.09	-0.12	-					
Empowering Managership	5.22	1.38	-0.12	0.01	0.19**	-0.02	**0.88**				
Work-Family Negative Spillover	2.24	0.71	0.02	0.12	0.10	0.06	-0.34**	**0.80**			
Work-Family Positive Spillover	4.14	0.84	-0.29**	0.04	.161*	0.02	0.44**	-0.16*	**0.73**		
POS (Level 2)	2.78	0.87	0.10	-0.01	-0.02	-0.01	-0.20**	0.05	-0.07	**0.73**	
Manager Turnover Intention	2.86	1.46	0.29**	-0.06	-0.06	-0.02	-0.28**	0.11	-0.37**	0.12	**0.77**

Note: N = 220

*p≤ .05

**p≤ .01; the square roots of the AVEs are in bold in the diagonal cells; POS = perceived organizational support

### 4.2 Measurement model

This study adopted a two-step approach [40] to the testing of the proposed model. The first step involved the use of SPSS 26.0 and AMOS 24.0 for the evaluation of the measurement model aimed at assessing the validity of the data. The second step involved the use of AMOS 24.0 and PROCESS macro 4.0 for the analysis of the structural model for the testing of the hypothesized relationships. POS was used as a level-2 variable (at the organizational level) in the model, and the EL, WFPS, WFNS and turnover intentions were treated as level-1 variables (at the individual level) [41].

A confirmatory factor analysis (CFA) was performed so as to test the validity and reliability of the data. The results in Table 2 show that the factor loadings of all the items were higher than the threshold of 0.60 [42], thus providing evidence of the validity of the measurement model. The convergent validity was assessed by calculating the Cronbach’s alpha, composite reliability, maximum shared variance (MSV) and Average Variance Extracted (AVE) values. The results revealed that the values ranging from 0.85 to 0.98 for the Cronbach’s alpha, from 0.85 to 0.98 for the composite reliability, and from 0.53 to 0.78 for the AVE values were all above the acceptable ranges [43]. Furthermore, the results revealed that all the MSV values were lower than the AVE values. The results thus provide evidence of the convergent validity of the measurement model. In addition, aimed at the testing of the discriminant validity of the measurement model, the square roots of the AVEs were calculated and compared with the corresponding correlations between the constructs. The good degree of the model fit indices provided a reasonable data fit for the testing of the structural model of the study. We determined values of CMIN/DF = 1.80, CFI = 0.92, TLI = 0.91, RMSEA = 0.06, all of which were above the threshold [44].

**Table 2:** Results of the confirmatory factor analysis.

Variable	CR	MSV	AVE	α	Factor loadings
Empowering Managership	0.98	0.20	0.78	0.98	0.82–0.94
Work-Family Negative Spillover	0.87	0.13	0.63	0.86	0.60–0.89
Work-Family Positive Spillover	0.93	0.20	0.54	0.93	0.60–0.81
Perceived Organizational Support	0.91	0.06	0.53	0.91	0.64–0.85
Manager Turnover Intention	0.85	0.18	0.59	0.85	0.65–0.85

Note: N = 220, CR = composite reliability, AVE = average variance extracted, α = Cronbach’s alpha.

### 4.3 Structural model

The results of the structural model analysis are shown in Table 3. Hypothesis 1 posited a negative association between EL and the manager WFNS. The results revealed a significant negative relationship between these variables (β = -0.19, SE = 0.03, p ≤ 0.001), thus supporting Hypothesis 1. In line with Hypothesis 2, which predicted a positive association between EL and the manager WFPS, the results provided evidence of a significant positive relationship between EL and WFPS (β = 0.24, SE = 0.04, p ≤ 0.001).

**Table 3:** Results of the testing of the hypotheses.

Outcome variable:	Work-Family Negative Spillover			Work-Family Positive Spillover			Work-Family Negative Spillover			Work-Family Positive Spillover		
	β	SE	t	β	SE	t	β	SE	t	β	SE	t
Gender	-0.05	0.09	-0.54	-0.43	0.10	-4.13***	-0.04	0.09	-0.40	-0.42	0.10	-4.08***
Age	0.10	0.07	1.46	0.04	0.08	0.55	0.09	0.07	1.26	0.03	0.08	0.37
Education	0.17	0.07	2.37*	0.10	0.08	1.29	0.19	0.07	2.72**	0.13	0.08	1.62
Organizational tenure	0.04	0.03	1.31	0.02	0.03	0.74	0.04	0.03	1.35	0.03	0.03	0.79
Empowering Managership	-0.19	0.03	-5.74***	0.24	0.04	6.56***	-0.19	0.03	-5.52***	0.25	0.04	6.68***
Perceived Organizational Support (Level 2)							0.01	0.05	0.27	-0.03	0.06	-0.52
Interaction effect							0.07	0.04	1.89	0.09	0.04	2.05*
Mediation effects												
IV	M			DV			β	SE	CI-LL	CI-UL		
Empowering Managership	Work-Family Negative Spillover			Manager Turnover Intention			-0.01	0.03	-0.07	0.04		
Empowering Managership	Work-Family Positive Spillover			Manager Turnover Intention			-0.10	0.03	-0.17	-0.04		

Note: N = 220

*p≤ .05

**p≤ .01, IV = independent variable, M = mediator, DV = dependent variable, CI-LL = 95% confidence interval lower limit, CI-UL = 95% confidence interval upper limit

The mediation hypotheses were tested applying the bootstrapping approach in order to calculate the confidence intervals for the indirect effects [45], an approach that is considered superior to traditional mediation analysis methods [46]. Hypothesis 3 proposed that the manager WFNS would act as a significant mediator in the relationship between EL and manager turnover intentions. The results shown in Table 3 do not support this hypothesis (β = -0.01, SE = 0.03, 95% CI [-0.07, 0.04]). In contrast, Hypothesis 4, which predicted the manager WFPS as a significant mediator in the relationship between EL and manager turnover intentions, was supported; the results indicated that WFPS is indeed a significant mediator in this relationship (β = -0.10, SE = 0.03, 95% CI [-0.17, -0.04]).

The study examined the moderating and moderation-mediation hypotheses applying the bootstrapping approach in PROCESS macro. Hypothesis 5 proposed that the level of POS moderates the relationship between EL and WFNS such that the relationship weakens with an increase in POS and is strengthened with a decrease in POS. The results presented in Table 3, however, did not support this hypothesis (β = 0.07, SE = 0.04). Accordingly, the hypothesis was not supported by the empirical data. In contrast, Hypothesis 6 predicted that the POS level moderates the relationship between EL and WFPS such that the relationship is strengthened with an increase in POS and weakens with a decrease in POS. The interaction effect presented in Table 3 provided evidence of the significant moderating role of the cross-level POS in the relationship between EL and WFPS (β = 0.09, SE = 0.04, p ≤ .05). Simple slope analysis was subsequently performed aimed at providing further support for the interaction effect.

**Fig 2:**
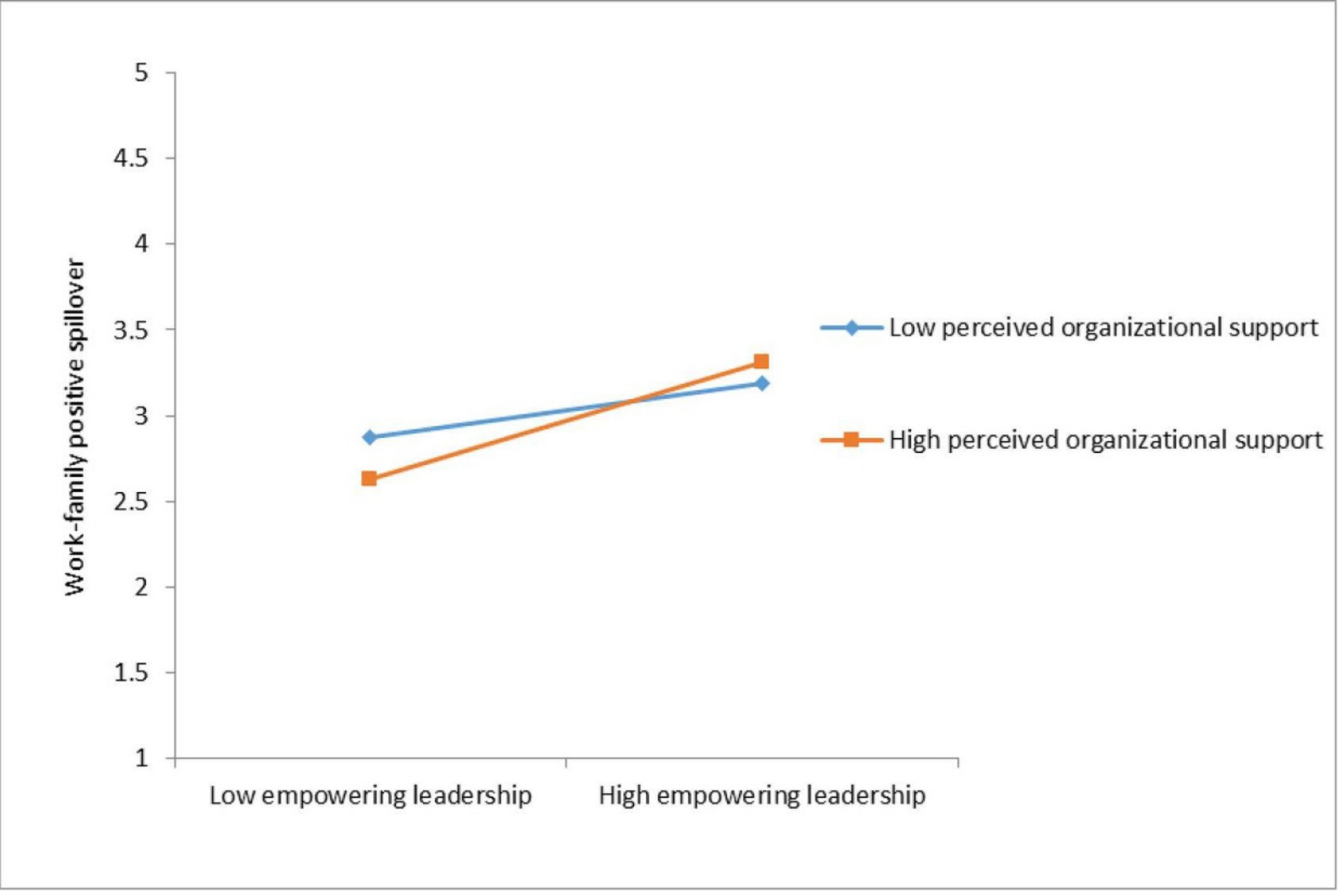
The interaction impact of empowering managership and perceived organizational support on work-family positive spillover.

The results of the moderation analysis at low and high POS levels are shown in Fig 2. A simple slope test was performed in order to validate the interaction effect. The results revealed that at high POS levels (one standard deviation above the mean), the impact of EL on WFPS was strongest (β = 0.33, SE = 0.06, p ≤ .001) as compared to the mean POS level (β = 0.25, SE = 0.04, p ≤ .001) and low POS levels (one standard deviation below the mean) (β = 0.18, SE = 0.05, p ≤ .001). These findings provide supporting evidence for Hypothesis 6.

The results of the analysis of the moderation and moderation mediation hypotheses, as presented in Table 4, do not support Hypothesis 7. This finding is not surprising since the previously reported insignificant mediation effect of the manager WFNS in the relationship between EL and manager turnover intentions and the insignificant moderation effect of POS on the relationship between EL and the manager WFNS were both considered. However, the results presented in Table 4 provide support for Hypothesis 8. The analysis revealed that the POS level exerts a significant moderating impact on the indirect relationship between EL and manager turnover intentions via WFPS. More specifically, the results indicate that this indirect relationship is strengthened at high POS levels (one standard deviation above the mean) (β = -0.14, SE = 0.05, 95% CI [-0.23, -0.05]) as compared to the mean POS level (β = -0.10, SE = 0.03, 95% CI [-0.17, -0.04]) and low POS levels (one standard deviation below the mean) (β = -0.07, SE = 0.03, 95% CI [-0.15, -0.02]).

**Table 4:** The conditional indirect impact of empowering managership on manager turnover intentions via mediating variables at the moderator level.

IV	M	DV	Moderator (Level2)	Values of moderator	Effect	SE	CI-LL	CI-UL
Empowering Managership	Work-Family Negative Spillover	Manager Turnover Intention	Perceived Organizational Support	-SD	-0.02	0.04	-0.09	0.06
Empowering Managership	Work-Family Negative Spillover	Manager Turnover Intention	Perceived Organizational Support	Mean	-0.01	0.03	-0.07	0.04
Empowering Managership	Work-Family Negative Spillover	Manager Turnover Intention	Perceived Organizational Support	+SD	-0.01	0.02	-0.05	0.03
Empowering Managership	Work-Family Positive Spillover	Manager Turnover Intention	Perceived Organizational Support	-SD	-0.07	0.03	-0.15	-0.02
Empowering Managership	Work-Family Positive Spillover	Manager Turnover Intention	Perceived Organizational Support	Mean	-0.10	0.03	-0.17	-0.04
Empowering Managership	Work-Family Positive Spillover	Manager Turnover Intention	Perceived Organizational Support	+SD	-0.14	0.05	-0.23	-0.05

Note: N = 220, IV = independent variable, M = mediator, DV = dependent variable, CI-LL = 95% confidence interval lower limit, CI-UL = 95% confidence interval upper limit.

## 5. Discussion

This study investigated the impact of EL on WFNS and WFPS and manager turnover intentions in the hospitality and tourism sectors via the testing of various hypotheses applying the bootstrapping approach in PROCESS macro. The results of the study revealed a significant positive relationship between EL and both the manager WFPS and WFNS, which supplements the existing expert literature by illuminating the role of EL in influencing managerial outcomes; previous studies have focused primarily on the relationship between various underlying mechanisms associated with managerial outcomes and employee behavior [47].

The results attained in our study revealed that POS plays an important role in terms of moderating the relationship between EL and both the manager WFPS and WFNS. This is in line with previous research that highlighted the significance of POS in manager-follower interactions [32]. In addition, the results of our study determined that the moderating effect of POS was evident in the indirect relationship between EL and manager turnover intentions via the manager WFPS, with a stronger indirect impact at higher POS levels.

Conversely, the study results revealed the insignificant mediating effect of the manager WFNS on the relationship between EL and manager turnover intentions and the insignificant moderation effect of POS on the indirect relationship between EL and manager turnover intentions via WFNS. These somewhat unexpected results suggest that the mediating role of the manager WFNS is not as significant as previously thought and that further research may be necessary in order to fully understand its role in the relationship between EL and manager turnover intentions.

Overall, the findings of this study contribute to the expert literature by providing new insights into the relationship between EL, the manager WFNS and WFPS and manager turnover intentions in the hospitality and tourism sectors. These results have implications for both theory and practice in terms of their potential to influence management strategies concerning the retention of managers and improving the organizational outcomes in these sectors.

### 5.1 Theoretical implications

This study makes several important contributions to the fields of hospitality and tourism. Firstly, the research enhances our understanding of the concepts of EL and work-family spillover via the application of the contextual resource framework of the COR theory. The integration of EL with work-family spillover (WFNS and WFPS) provides a new perspective in terms of the application of EL in the context of the work-family balance of managers. The categorization of EL as a contextual work resource provides managers with greater flexibility and confidence, which allow them to more effectively achieve their personal and professional objectives. Our study contributes to addressing a gap in the literature by examining the direct impact of EL on managers as a response to the limited research that has been conducted to date on the effects of various leadership approaches to the work-family balance of managers [48]. In addition, the findings of the study provide the foundations for further research in this area.

Our study expands the existing literature by making several important theoretical contributions to issues in the hospitality and tourism industry. Firstly, the research enhances our understanding of the relationship between EL and work-family spillover by explicitly defining the scope of the COR theory concerning work-family spillover in terms of leadership. By integrating EL with WFNS and WFPS, the study enhances both experimentally and conceptually, our understanding of the impact of EL on the work-family balance of hospitality and tourism sector managers. In line with the COR framework, EL is classified as a contextual work resource that enables managers to balance their personal and professional lives by providing them with the necessary flexibility and confidence to overcome barriers to performance and to achieve their goals. In line with research [49], the study expands the examination of leadership attitudes and their impacts on the work-family balance of employees by exploring the influence of EL in this context.

A number of other studies have explored the relationship between leadership style and the work-family interface of followers. Transformational and authentic leadership perspectives have been found to be beneficial for the work-family interface [8, 50], while servant leadership has been associated with work-family enrichment [51]. Conversely, abusive supervision has been linked to work-family conflict and family destabilization [3]. Li and colleagues [9] included empowerment in their conceptual model of the influence of leadership behavior on work-family interaction. However, there remains the need for further research aimed at expanding our understanding of the impact of leadership approaches on the compatibility of work and family for employees in the hospitality and tourism sector. This study represents a significant step forward in this respect by exploring the role of EL in this relationship.

Our study further provides a significant contribution to the existing expert literature by examining the impact of EL on work-family spillover (both WFNS and WFPS) applying the resource conservation framework. While previous studies have explored the effects of EL on work outcomes [11, 52], our study expands such research by examining the spillover effect of EL beyond the workplace and its impact on the work-family balance. In terms of the resource conservation theory, this study explains the unique links between EL and the intentions of managers to leave their positions via the spillovers that affect the family labor of employees. The results of our study provide an ovel insight into the role of EL in terms of enhancing the relationship between the work and family domains, and pave the way for future research on the relationship between EL and manager turnover intentions via other underlying mechanisms, e.g. stress. Given the scarcity of research in this area to date, we expect that this study, in conjunction with a previous seminal work [9], will stimulate further research on EL across a range of settings, thus resulting in a deeper understanding of its impact on the work-family balance and leadership outcomes.

Our study contributes to the existing literature on the intersection of leadership and work-family dynamics by focusing on the delegation of control or authority as a key mechanism for improving the work-life balance of employees. Previous research has synthesized the various leadership styles that promote moral leadership, including serving, transformational, authentic and ethical leadership [53]. However, this study goes beyond these theories by specifically highlighting the role of EL as a unique leadership approach that differs from other styles by distributing control and power to followers [54]. A recent meta-analysis has shown that EL is positively associated with social capital, in a similar way to transformational leadership, and that it is effective in terms of fostering innovation and proactivity [31]. Our findings respond to the call for the consideration of EL in leadership studies [12] and provide valuable insight for managers seeking to introduce EL approaches.

Thirdly, our study advances the literature on leadership by exploring the connection between the empowering behavior of managers and their intentions to leave their position, as posited by the Conservation and COR theories, suggesting that individual resources play a mediating role [55]. The findings of the study revealed the relationship between workplace resources and work-family spillover via two mediators, i.e. social capital resources and self-efficacy [56]. Our results provide a more nuanced view than do previous studies that focused merely on a single mediator [57]. This study underscores the significance of incorporating both WFNS and WFPS as mediators in the relationship between EL and turnover intentions. The findings of the study have the potential to serve as a valuable resource for management scholars seeking to enhance their comprehension of the links between work resources and the work-family balance in the tourism industry. Fourthly, our study provides new insight into the mediating role of work-family spillover and the moderating influence of POS on the relationship between EL and manager turnover intentions. This contribution is significant since previous research on work-family models has relied primarily on the boundary theory to explain the reduced impact of leadership on the work-family balance of employees [58, 59]. Our findings suggest that POS is also a crucial factor in terms of mitigating the effect of leadership on work-family interactions. POS refers to the extent to which managers identify with and internalize organizational values and goals, which enhances their sense of power as agents of the organization [60]. Our study employed a time-lag approach and multilevel analysis in order to assess the effect of EL on the ability of managers to balance work and family, and performed a moderated mediation analysis aimed at examining the influence of POS on WFNS and WFPS in the hospitality and tourism industry.

### 5.2 Practical implications

This study has both practical and theoretical implications. Interest has grown substantially in recent years in terms of exploring the relationship between work and family life [61], and organizations and managers have adopted various measures aimed at supporting employees in their quest to balance their work and family obligations [62]. On the practical side, this research provides a useful strategy for managers who are seeking to empower their employees and reduce stress levels, while also benefiting in terms of their own work-family balance. On the theoretical side, our study highlights the relationship between EL and behavioral approaches such as valuing the work of employees, promoting discretion in decision-making, expressing confidence in employee abilities and removing barriers to effectiveness [63].

We recommend that companies in the hospitality and tourism sectors provide EL training for managers aimed at promoting the delegation of responsibilities to employees. Furthermore, training and education are required to improve the understanding of how EL approaches are able to benefit managers themselves. Such training should encompass concepts such as autonomy, equality, collaborative decision-making and leadership by example. Previous research indicates that EL behavior is fostered by factors such as trust and humility, while power distance and uncertainty avoidance represent obstacles in this respect [64]. Therefore, organizations should strive to cultivate and maintain qualities such as trust, humility, egalitarianism and risk-taking in their leadership approach.

This study highlights the significance of leadership and organizational support with respect to optimizing the perceived benefits of EL. It also underscores the need to raise the awareness of managers of the importance of delegating authority to subordinates, which has both positive and negative consequences for executives. Hence, it is crucial for managers to be aware of how EL approaches are able to support their own work-family balance.

### 5.3 Future research avenues and limitations

This study has a number of strengths, including the use of the time-lag approach for the sampling and data collection. However, there are also several limitations to be considered when interpreting the results. Firstly, the constructs in this study are self-reported, which raises issues concerning the potential for common method bias, the potential for which is, however, mitigated to some extent by the use of the time-lag approach, i.e. a time-wave research method. The statistical analysis indicated that common method bias is not a major concern in this study. Moreover, it has been acknowledged in the field of work-family studies that such assessments are subjective and may be difficult to measure accurately. Aimed at enhancing the validity of the results, future studies should adopt a multi-source approach, include samples from multiple cultures and incorporate additional variables.

Secondly, the absence of a causal relationship between the EL variable and the two mediators WFPS and WFNS comprises a further limitation. Although the data for EL and the mediators was collected at different time points, it was not possible to establish a causal relationship. The expert literature suggests that WFPS and WFNS tend to support empowerment and that managers with a strong WFPS and WFNS are able to empower their followers. Further research applying a longitudinal or time-lag approach would assist in the understanding of the causal relationships between EL, WFPS and WFNS; such approaches would allow for a more comprehensive understanding of these relationships.

Thirdly, the findings of the study have limited potential for generalizability since the data was collected exclusively from organizations active in the Chinese hospitality and tourism sector. The cultural context of this study is crucial given that Chinese society is characterized by a high prevalence of collectivism, traditional values and power distance [65]. Previous research has shown that Leader-Member Exchange (LMX) is moderated in collectivist societies in a manner that acts to weaken the correlation between leadership trust and outcome variables, as compared to individualistic contexts [66]. It is worthy of note that individuals in collectivist cultures are heavily influenced by the behavior of their managers and by the reciprocal responsibilities and roles demanded of them [67]. Furthermore, traditional values have been shown to be in conflict with the self-efficacy benefits of delegation since employees with strong traditionalist values often fear empowerment and responsibility [68].

A meta-analysis [69] determined a significant association between EL and social capital. The expert literature and empirical evidence suggest that the relationship between EL and work-family spillover may be stronger in Western than in Chinese samples [17]. Moreover, this study indicates that the moderating effect of POS may be more pronounced among women. Our finding of the non-significant mediating impacts of WFPS and WFNS on the connection between EL and manager turnover under significant POS may not be applicable in Western contexts due to such cultural variations. Therefore, future research would benefit from the consideration of cross-cultural or cross-industry samples aimed at supplementing the findings presented herein.

A further limitation of our study concerns the restricted focus on lower and mid-level managers and the consequent absence of input from employees and top-level executives. Since the study relies solely on self-reported responses from these managers, it is worth noting that previous research has indicated that individuals may have differing perceptions of work-family spillover [70]. This study employs "ratings" as a means of obtaining manager feedback in the work-family domain. Nevertheless, it is vital that we recognize that both followers and top management exert significant impacts on the resources available for handling work-family conflicts. Consequently, future research in the hospitality and tourism industry should consider the role of organizational support in terms of managing work-family spillover and turnover intentions.

## 6. Conclusion

The study employed a time-lag approach in a multi-level analysis aimed at evaluating a conceptual model that explores the impact of EL on work-family spillover and manager turnover intentions in the hospitality and tourism industry. Our findings revealed that when work-family spillover is moderated by POS, EL perceptions exert no significant impact on WFNS. Moreover, our results indicated that the impact of EL on work-family spillover is greater for employees with high levels of WFPS than for those with low levels of WFNS. A theory-based model based on COR was applied so as to examine the impacts of work and family in this context, the results of which indicate the need for further research on the effects of EL on both businesses and households.
