# Correction: Turning the tide on turnover: The impact of empowering leadership on the work-family spillover of managers

**DOI:** 10.1371/journal.pone.0347579

**Published:** 2026-04-16

**Authors:** Naseer Abbas Khan, Waseem Bahadur, Robin Maialeh, Natayla Pravdina, Maria Akhtar

There are errors in Fig 1. Please see the correct Fig 1 here.

**Fig 1:**
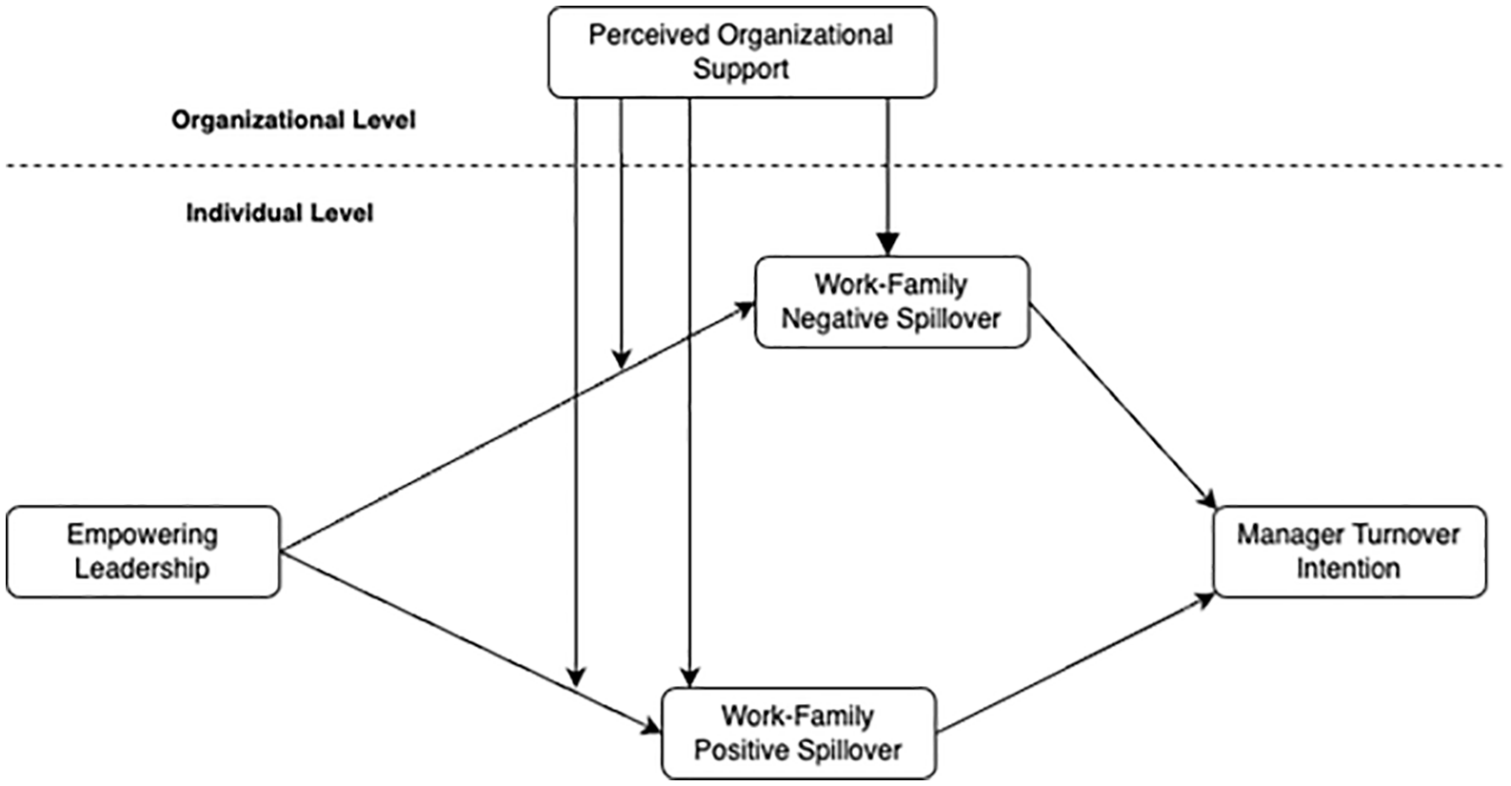
Research model.
